# Fundamentals of big data and artificial intelligence in transfusion medicine

**DOI:** 10.1111/vox.70227

**Published:** 2026-03-23

**Authors:** Amin T. Turki, Christian Martin Brieske, Umut A. Gurkan, Katja M. Scheidler, O. Berk Usta, Esa Turkulainen, Kamyar Arzideh, Christian Temme, René Hosch, Peter A. Horn, Mikko Arvas

**Affiliations:** ^1^ Computational Hematology Lab, Institute for Artificial Intelligence in Medicine University Hospital Essen Essen Germany; ^2^ Institute for Artificial Intelligence in Medicine University Hospital Essen Essen Germany; ^3^ Department of Hematology and Oncology, University Hospital Marienhospital Ruhr‐University Bochum Bochum Germany; ^4^ Institute for Transfusion Medicine University Hospital Essen Essen Germany; ^5^ Department of Mechanical and Aerospace Engineering Case Western Reserve University Cleveland Ohio USA; ^6^ Center for Engineering in Medicine & Surgery, Massachusetts General Hospital Harvard Medical School Boston Massachusetts USA; ^7^ Research and Development Finnish Red Cross Blood Service Helsinki Finland

**Keywords:** AI, biobank, crossmatch, decision support, FHIR, lab‐on‐a‐chip

## Abstract

Within the digital transformation of medicine, transfusion medicine has quietly become a big‐data discipline. The long‐standing tradition of blood product standardization (e.g., ISBT‐128) and large donor cohorts being followed over years—some of which are sampled in national biobank projects, build a favourable setting. In parallel, recent advances in artificial intelligence (AI) and data integration facilitate efficient data use for research and clinical care. Consequently, next‐generation blood services might monitor donor phenotype data and match this information to AI‐predicted recipient demands and their outcomes. Here, we attempt to provide a comprehensive introduction to the possibilities and challenges of big data and AI in transfusion medicine along with data integration opportunities related to the Fast Healthcare Interoperability Resources standard. We educate on the principles of AI and the digital transformation of transfusion medicine and analyse the evidence of blood establishments as digital platforms. We illustrate possible roadmaps for data integration and how federated learning initiatives and national networks may scale value while preserving donor and patient privacy. Finally, we exemplify the ongoing transformation with precision red blood cell (RBC) diagnostics using lab‐on‐a‐chip and the digital crossmatch. The practice of transfusion medicine is undergoing transformation and experimentally appears to profit from synergies in precision diagnostics and AI. Its translation into routine practice remains a challenge for the current decade to leverage the full potential of blood establishments as ‘big‐data engines’.

**Highlights**

- Transfusion medicine is silently becoming a big data discipline with substantial potential for artificial intelligence methods and applications.
- This transformation requires post‐graduate education in the relevant principles for clinical application.
- The synergy of precision red blood cell diagnostics (e.g., via ‘lab‐on‐a chip’) and harmonized electronic health record data for personalized blood transfusions is substantial.

## BIG DATA AND ARTIFICIAL INTELLIGENCE IN TRANSFUSION MEDICINE: BASIC UNDERSTANDING AND DOMAINS OF APPLICATIONS

Globally, cellular therapies attract increasing interest and activities across medical disciplines, yet their oldest and most commonly used form is haemotherapy, under the supervision of transfusion medicine specialists. Their experiences, standards (e.g., ISBT‐128) and cell manipulation resources in blood establishments are critical for a streamlined therapy pipeline and for the future growth of cellular therapy practices. In parallel, transfusion medicine has quietly evolved into a big‐data discipline. Each step of the ‘vein‐to‐vein’ chain—from donor recruitment and testing to collection and processing, inventory management, allocation, bedside transfusion and post‐transfusion monitoring—generates extensive, heterogeneous data [1]. These include donor demographics and baseline laboratory results; timestamps and device metadata from collection procedures; manufacturing and quality‐control metrics from processing; allocation and logistics records from inventory; and transfusion histories and clinical outcomes on the patient side. Blood establishment computer software systems are used to manage these processes. Increasingly, such operational data streams are enriched with collective molecular quantification information (OMICs) [2], questionnaire data, medical device outputs and haemovigilance reports. Taken together, they form an exceptionally dense digital footprint of what has traditionally been routine practice. The regulatory framework for blood products is, and continues to be, highly strict in both Europe and the United States, reflecting their status as perishable human‐derived products with direct implications for patient safety [3].

Within these constraints, artificial intelligence (AI) methods are beginning to make an impact [4]. Predictive AI models and natural language processing can potentially support donor safety prediction, anomaly detection, phenotype modelling of stored red cells, demand forecasting and the digitization of free‐text reports. For such applications to work across institutions, interoperability standards are critical. The Fast Healthcare Interoperability Resources (FHIR) allow data to be represented in an interoperable format, across different electronic health record (eHR) systems, while national initiatives, for instance FinOMOP, seek to harmonize countrywide datasets using the Observational Medical Outcomes Partnership common data model. Without such standards, the growing volume of data risks creating fragmentation rather than insight; with them, transfusion medicine is uniquely well placed to convert data into clinical value.

**FIGURE 1:**
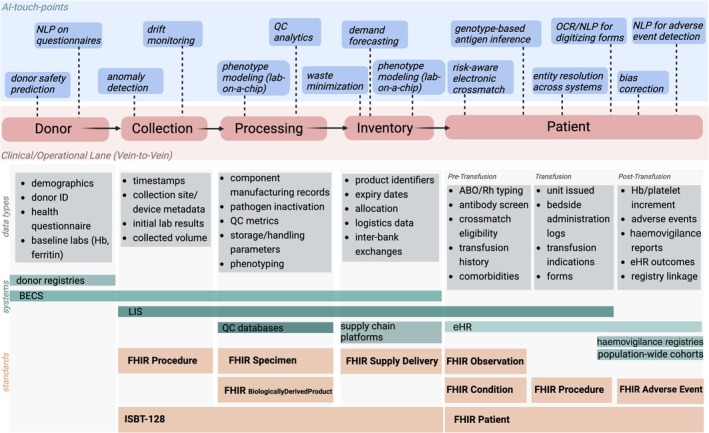
Illustrative sketch of transfusion medicine data flows. The transfusion medicine keychain of donor, collection, processing, inventory and patients marks the centre of this illustration, which integrates both artificial intelligence (AI) touchpoints (above, blue), data types (below, grey), systems and standards such as Fast Healthcare Interoperability Resources (FHIR) and those of ISBT. Collection and cell processing facilities are integrated, as well as hospitals or private practices. BECS, Blood Establishment Computer System; ID, identifier or pseudonym; eHR, electronic health record; Hb, haemoglobin; LIS, laboratory information system; NLP, natural language processing; OCR, optical character recognition; QC, quality control.

This shift is illustrated in Figure 1, which maps transfusion medicine process chain with the corresponding data types, clinical systems, interoperability standards and AI ‘touchpoints’. The figure highlights how blood services are no longer merely suppliers of products but are evolving into population‐scale data platforms. By enabling structured links between donor, product and patient outcomes, they create an ecosystem where routine service data can also serve as a research and innovation resource. Population cohorts bring this platform's potential into focus. The Danish Blood Donor Study (DBDS) [5], for example, integrates questionnaire, laboratory and genotype data with national health registries, building a longitudinal infrastructure that supports both biomedical discovery and service improvement. Similar initiatives illustrate how donor cohorts and registries can scale to the national level and connect with broader biobank efforts. Together, these initiatives demonstrate that blood establishment could develop into active contributors of precision healthcare and translational research. By linking donor characteristics to product quality and patient outcomes, that is, building vein‐to‐vein databases, they could operate population‐scale ‘learning loops’: donor information informs product characteristics, which in turn link to patient outcomes, and these outcomes then feed back into donor selection and processing standards. With governance and interoperability in place, this cycle can continuously refine safety, logistics and personalized compatibility, effectively transforming transfusion services into living laboratories for clinical innovation.

This review provides an educative narrative and exemplifies, for example, the use of lab‐on‐a‐chip testing along with data integration opportunities related to the FHIR standard in hospitals. We provide a brief technical introduction to the core concepts of big data and AI most relevant to transfusion medicine specialists. We emphasize the achievements of large cohort initiatives, the challenges of data integration and the opportunities that arise when AI is embedded into transfusion workflows. To make the discussion concrete, we highlight three frontier use cases: (1) Precision medicine red blood cell (RBC) quality assessment, where phenotyping and AI can move beyond simple storage‐age proxies; (2) the emergence of digital crossmatching as a scalable alternative to serological compatibility testing and (3) AI support for estimating patient‐centred immunization probability and forecasting platelet demand.

**FIGURE 2:**
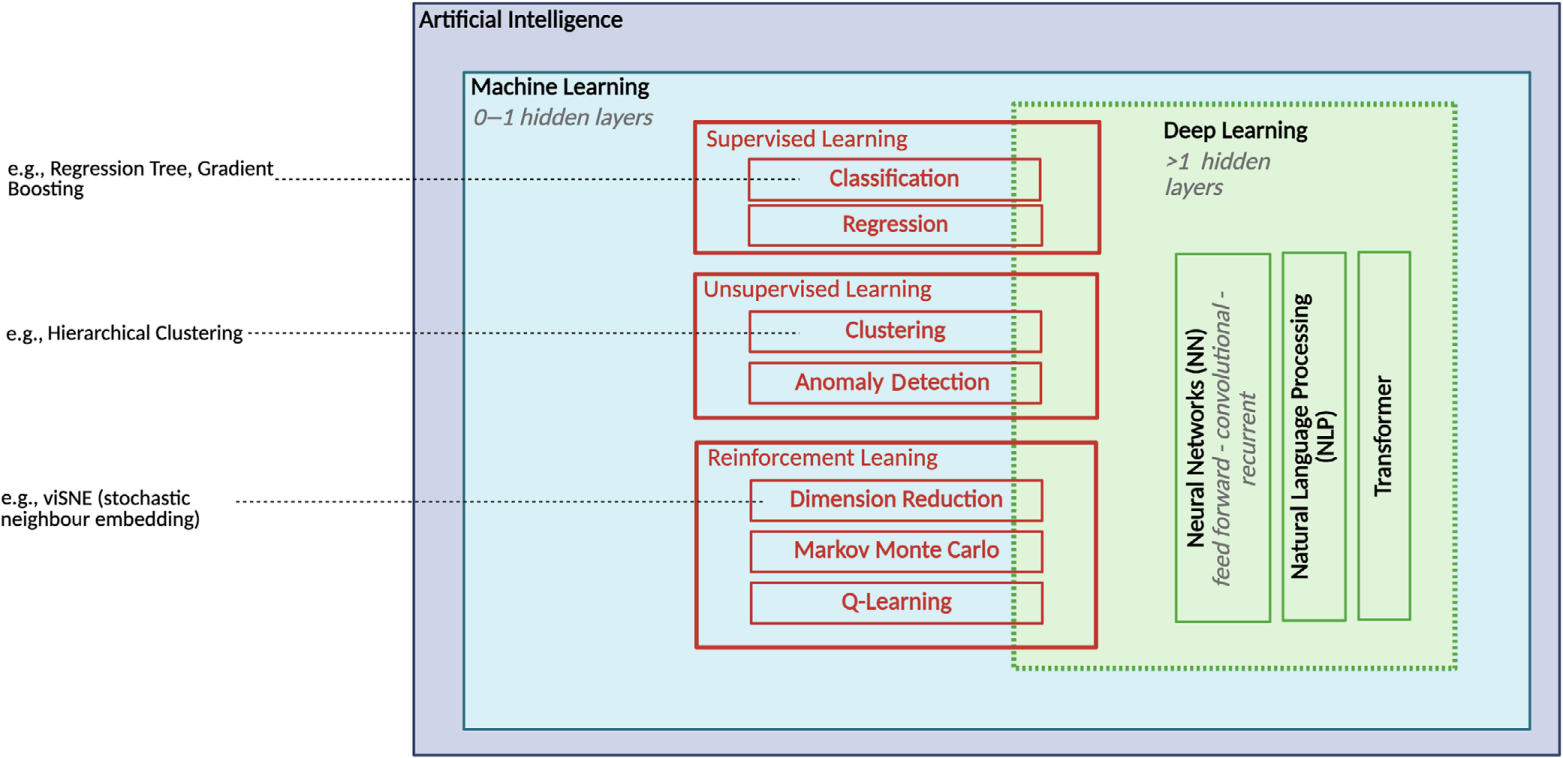
Overview of artificial intelligence paradigms relevant to transfusion medicine. Artificial intelligence (AI) encompasses several paradigms, each with distinct applications, advantages, and limitations. Supporting Information provides further details on respective AI methods.

**FIGURE 3:**
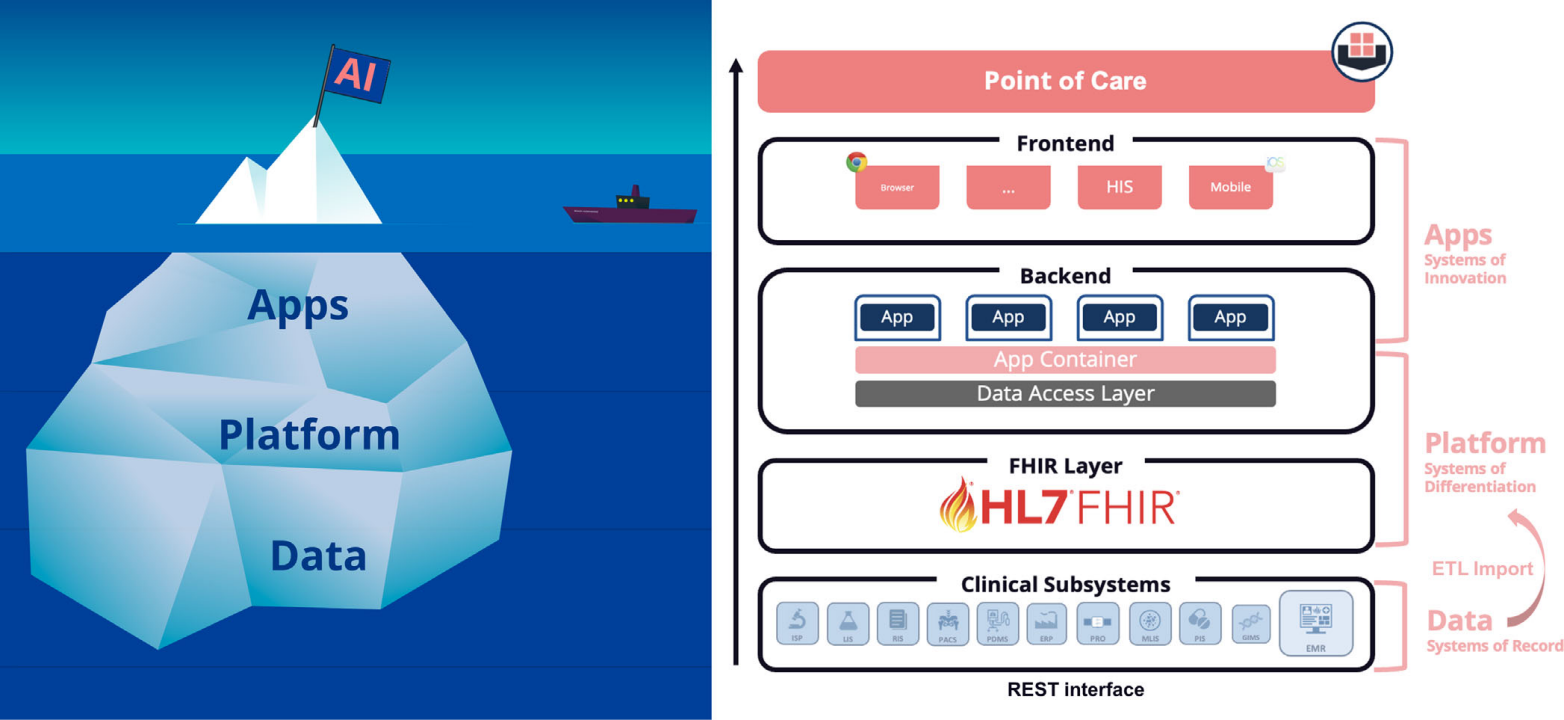
Sketch of the relationship between large datasets, platforms and artificial intelligence (AI) tools in hospital and care environments (left). Data integration infrastructure at the University Medicine Essen (right). EMR, electronic medical records; ETL, extract, transform, load; FHIR, Fast Healthcare Interoperability Resources; GIMS, Genetic Information Management System; HIS, hospital information system; ISP, Interoperability Standards Platform; LIS, laboratory information system; PACS, Picture Archiving and Communication System; PRO, patient reported outcomes; REST, representational state transfer.

The anticipated rise of AI in this domain raises the demand for postgraduate education and technical literacy within the community. To this end, the following section introduces the fundamental AI paradigms: predictive modelling, rule‐based decision support, unsupervised learning and reinforcement learning to explore their relevance for transfusion medicine (Figure 2). The first paradigm to consider is predictive modelling. By leveraging large datasets, predictive models can uncover risk factors and patterns that enable earlier interventions and more personalized treatment plans. In practice, they are used to predict disease progression, remission rates or the likelihood of patient deterioration, thereby supporting timely responses and improving operational efficiency. A further advantage lies in risk stratification: patients can be ranked by predicted risk, ensuring that limited resources are directed to those who will benefit most. At the same time, predictive models exemplify many of the challenges that come with AI in medicine. Their performance depends heavily on data quality and completeness, and systematic biases such as the underrepresentation of specific populations can result in inequitable care. Ethical concerns also arise around patient privacy and the secondary use of sensitive data. From a technical standpoint, embedding predictive models into existing hospital information technology systems is complex and resource‐intensive (Figure 3). Another central limitation is interpretability; many predictive models operate as ‘black boxes’, producing outputs without understandable reasoning. This may potentially erode trust or risk over‐reliance at the expense of clinical judgement and individualized patient care. Rule‐based decision support represents a contrasting approach. These systems are valued for their transparency because clinicians can easily trace recommendations back to evidence or logic. They have proven their worth in settings requiring rapid, standardized decisions and remain closely aligned with many needs of transfusion medicine. Yet they are inflexible, require continuous updating, and often depend on clean, structured inputs rarely available in real‐world healthcare data. Without maintenance, they can become outdated and fail to capture the complexity of atypical cases.

Machine learning (ML) approaches offer greater adaptability. Supervised methods are applied when a labelled outcome is known (Figure 2), for example, to estimate transfusion response or predict complications, while unsupervised methods can uncover hidden structures in heterogeneous datasets such as multiomics, immune profiles or eHRs. Reinforcement learning illustrates the potential of algorithms that optimize sequential decision‐making, for instance, in inventory management. Despite their differences, both rule‐based and ML approaches share limitations. Neither adequately accounts for the individuality of the healthcare professional who must ultimately use them, and neither adapts how knowledge is presented. Even accurate predictions may fail to deliver their full benefit if they are not communicated in a way that matches the user's context. Finally, there is the risk of over‐reliance on models, potentially overshadowing clinical judgement and individualized patient care [6].

Technical limitations include model accuracy, which can be affected by the quality and completeness of data. Biases in data, such as the underrepresentation of certain populations, can lead to inequitable care [7]. Ethical concerns arise regarding patient privacy and predictive data use in clinical decisions. Additionally, integrating predictive models into existing digital healthcare infrastructure is complex and resource‐intensive, requiring significant investment in technology and training. In recent years, however, these paradigms have begun to converge, driven by the rise of large language models (LLMs) and foundation models. These can integrate the predictive accuracy of ML with the structured reasoning of rule‐based systems. By processing both structured and unstructured data, they provide a more comprehensive view of patient information and can tailor recommendations to the user's level of training or workflow. Their natural language interfaces allow outputs to be more interpretable, partly addressing the ‘black box’ concern. Yet, LLMs also bring risks such as hallucinations, making grounding in verified data and human oversight essential. Still, the interest of the transfusion medicine community in these tools is increasing [8]. For transfusion medicine, this convergence is potentially transformative. It opens the possibility of decision support that is not only accurate and evidence‐based but also adaptive to the complexity of real‐world data and to the needs of individual clinicians. By making heterogeneous information both comprehensible and actionable, AI has the potential to enhance the efficiency of transfusion services, reduce risks and ultimately improve patient outcomes. In the next section we will exploit the role of registries in the digital transformation.

## TRANSFUSION MEDICINE DEPARTMENTS AND BLOOD ESTABLISHMENTS AS RESOURCES FOR BIG DATA RESEARCH AND CARE INITIATIVES WITH DATA INTEGRATION

A transfusion starts from the collection of the required biological material, that is, blood collection. As a perishable human derived product, the characteristics of the donor, collection system and logistics affect patient outcomes, haemoglobin (Hb) incrementing, platelet incrementing or adverse transfusion effects. Many aspects of blood collection are logged, creating rich datasets of donation timestamps, Hb values, collection sites and recipient hospitals, sometimes supplemented by donor health questionnaires, additional laboratory measurements, donor demographics and product quality data. Unfortunately, even within Europe the ways in which blood collection is organized varies from very centralized national systems (e.g., France, Finland, the Netherlands) to a mix of blood collectors, as in Germany, or mostly hospital‐based collection like in Sweden. This variation creates equally diverse data collection policies, storage solutions and interpretations of the General Data Protection Regulation (GDPR), decreasing the conformity and the usability of the data. Currently, the state‐of‐the‐art approaches to analyse data across multiple blood collectors either involve combining data by anonymizing and aggregating it using containerized software [9] or simply sharing a script [10] that carries out the modelling task locally at each data controller. To govern the running of such collaborative analysis, blood donation researchers have formed the ‘SanguinStats’ platform [9]. However, modelling standardization will only get us so far, as the data collected by blood collectors is heavily shaped by their collection practices, local donor population and its genetics. Drawing generally applicable conclusions by studying just one blood collector is hence hard or impossible. Furthermore, because blood collectors strive to minimize negative outcomes of blood donation, for example, anaemia and fainting, data from donor cohorts tend to exhibit a lot of bias. To mitigate these issues, efforts in data consolidation and comparison across collectors or countries are highly recommendable, even just to show that variation in local practices can still produce very similar end results [11].

The promise of blood collection as a data resource for transfusion medicine (whether nationally centralized or internationally collaborative) can be further realized by the use and production of large cohort and register studies like FinnGen [12], INTERVAL [13], SCANDAT [14] and Recipient Epidemiology and Donor Evaluation Study (REDS), as well as biobanks like the Danish Blood Donor Study [5], the UK Biobank [15] and the Blood Service Biobank [16]. These allow for the integration of additional data types such as omics, additional laboratory measurements and other eHRs with either blood donor or transfusion recipient data. For example, genomics has been shown to be useful for selecting blood donors with respect to their health [17]. Furthermore, omics technologies are increasingly transforming our understanding of RBC storage and efficacy by enabling a deep characterization of their metabolism. Large‐scale genomic studies have identified multiple loci associated with haemolysis during storage [18]. Combinations of genomics, proteomics and metabolomic analysis propose that polymorphisms, for example, in genes associated to urate [19], kynurenine [20] and glycolysis [21], explain donor‐dependent variability in RBC efficacy. Also, omics profiling has revealed how caffeine disrupts RBC storage quality [22]. Together, integrated omics offers a pathway towards personalized transfusion medicine by predicting unit quality, guiding donor selection and improving post‐transfusion outcomes. Still, when moving into generalizations about the general population, the use of these data sources require thoughtful methodology. Notably, because veteran blood donors tend to be healthier than the general population, they introduce strong membership bias (healthy donor effect) to these datasets [23]. This selection bias can be seen also at the genetic level, as veteran blood donors are likely to have less genetic risk for anaemias, infections and mental disease [16]. As such, any studied outcomes that also affect the likelihood of a person being a blood donor run the risk of introducing collider bias. However, this is also an opportunity to study of the interactions of genetic variants in ‘very healthy’ populations.

Finally, to fully utilize the data collected by blood services and biobanking efforts for transfusion medicine, they need to be combined with data about transfusion events. Current literature shows that the so‐called ‘vein‐to‐vein’ databases (e.g., SCANDAT) can already improve our understanding of transfusion outcomes by linking donors with transfusion recipients [24]. Both the UK Biobank and FinnGen contain genotyping data for around half a million persons, many of whom have hospital episodes and have received blood products. Nevertheless, the largest published analysis of single‐unit RBC transfusions with blood donor genotypes and blood recipient eHR, to enable modelling of transfusion effects, comprises some 6000 events [25]. Similar analyses of interactions of patient genetics and blood transfusion effects are lacking, partly because large, detailed datasets of transfusion events have not been compiled. For example, in the United Kingdom, hospital procedures are recorded with the Classification of Interventions and Procedures (OPCS‐4) system, in which blood transfusions are recorded only if the patient was admitted solely for a blood transfusion. Similarly, in FinnGen, records of transfusion events are sporadic. Even with direct access to full eHR data, the actual reason for transfusion or compliance to guidelines is rarely collected by current systems, forcing researchers to create rule‐based, artificially derived reasons [26]. A notable exception is the electronic clinical decision support system (CDSS) developed in Oxford (UK) where each order is automatically checked against national guidelines, and a reason for making an uncompliant order needs to be specified [27]. Such tools may also support patient blood management and contribute, for example, to the discussion around liberal blood product use in high‐income countries with data of one million patients [28]. Also in this context, transfusion practices vary between countries, hospitals and medical specialties. When considering the usefulness of eHR transfusion data, the data‐generating process can greatly affect the studied outcomes. To this end, the Pan‐European Transfusion Research infrastructure (PETRA) [29] has been initiated to ultimately allow for high‐quality comparisons in transfusion practices across Europe. If the results from these initiatives are further combined with biobanking efforts that contain the rich omics data, eHR data could realize the full potential of blood collection records and biobanks for transfusion medicine.

## THE CHALLENGES OF BIG DATA AND DATA INTEGRATION AND THE RESPECTIVE SOLUTIONS OFFERED BY AI

Transfusion medicine is a highly interconnected specialty, both logistically and clinically. Blood products circulate across hospital departments—from emergency to operating rooms and between regional blood establishments, which must constantly exchange reserves and update inventories to meet fluctuating demands. For a single patient, transfusion decisions require an orchestration of data—bedside blood typing, antibody screening, cross‐matching and records of prior transfusions that may originate from external centres (Figure 1). The result is a vast body of heterogeneous data, fragmented across institutions and formats. Reliable integration of these streams is a major challenge.

The digital transformation of transfusion medicine is accompanied by a set of challenges that must be overcome to realize the potential of data‐driven and AI‐enabled services (Table S1). The most pressing difficulties arise from the sheer heterogeneity and fragmentation of data. Yet, FHIR has emerged as the standard interoperability resource for data integration. Implementing eHR as an FHIR server typically entails exposing standardized RESTful endpoints (often to eHR software, vendor‐provided) with SMART‐based authorization to enable interoperable application integration and data exchange. Alternatively, if the vendor does not provide these endpoints, the database of the eHR software can be accessed to transform the eHR data into FHIR. Formatting or transforming data in the FHIR standard most commonly follows an ETL (extract, transform, load) process from the eHR into a separate FHIR repository, although native persistence and event‐based messaging are also used. While FHIR offers a reusable, resource‐oriented model, significant barriers to collective analysis remain. These include incomplete resource coverage, the abundance of unstructured data (e.g., in medical notes) and persistent semantic heterogeneity despite structural standardization. Furthermore, large‐scale analytics generally require FHIR bulk data export, which introduces complex governance and audit trail requirements. For FHIR resources, which greatly facilitate the implementation of AI tools in hospitals, Hosch et al. [30] introduced FHIR‐PYrate, a Python package designed to facilitate data extraction from FHIR servers in a data science–friendly manner. This package simplifies querying and retrieving healthcare information, enabling efficient access to standardized medical data for analysis and research. By streamlining the process of interacting with FHIR servers, FHIR‐PYrate enhances the capability of researchers and data scientists to extract and utilize clinical data effectively.

Still, as discussed above, the required data is fragmented and differentially stored across Europe. Without harmonization, large‐scale comparative analyses remain constrained. AI methods can contribute to resolving this problem by using natural language processing and ontology‐mapping techniques to reconcile unstructured clinical notes with structured datasets, while representation learning methods help align heterogeneous data streams [31]. In combination with international standards such as ISBT‐128 and HL7 FHIR, AI‐based harmonization reduces the need for extensive manual curation and creates the technical foundation for cross‐institutional integration.

A second obstacle relates to data quality, bias and missingness as well as model transparency in data processing. Frequently, preprocessing techniques are insufficiently described in scientific publications, which may hinder the reproducibility of AI models. These differences in preprocessing can introduce bias that are hard to detect and may lead to hidden overfitting. Donation and transfusion datasets frequently suffer from measurement inconsistencies, selective underreporting and population‐specific biases, for example, ‘healthy donor effect’. Furthermore, transfusion events are often poorly coded in hospital information systems, obscuring the link between donor and recipient outcomes. AI methods can alleviate these issues through predictive imputation (i.e., filling missing data, by predicting missing values via a regression model and filling these with similar actual values), anomaly detection, and fairness‐aware learning algorithms, which not only identify implausible entries but also correct for systematic biases. Such methods can stress‐test findings before clinical translation and improve the reliability of transfusion data across populations.

Equally significant are the governance and privacy constraints associated with donor and recipient data, which are among the most tightly regulated in medicine. Here, an AI approach using federated learning allows to train models locally at each hospital or blood establishment, with only model parameters exchanged instead of raw data. This principle preserves privacy while still enabling collaborative research at scale. Emerging approaches such as secure multi‐party computation and differential privacy further enhance compliance while ensuring analytical power.

The operational context of transfusion medicine also demands scalability and real‐time applicability. Blood services must not only analyse retrospective datasets but also provide predictive insights in daily practice, for instance in inventory management, donor safety monitoring or patient compatibility matching. AI algorithms designed for online or reinforcement learning can continuously adapt to shifting demand patterns and donor inflow.

Finally, interpretability and clinical trust remain central barriers to adoption. Transfusion medicine specialists must be able to understand and contextualize AI‐driven insights if these are to inform clinical decisions. ‘Black box’ predictions without transparency undermine trust and may never be integrated into practice. Explainable AI techniques can mitigate this issue by highlighting the clinical features that drive predictions, thus linking model outputs with familiar parameters such as donor age, storage duration or recipient comorbidities. When combined with existing rule‐based decision support systems, this fosters accountability and ensures that AI functions as an adjunct rather than a replacement for clinical expertise. While ethical issues may also arise from the use of AI, these are reviewed elsewhere [32] and are not specifically covered by this review.

These challenges illustrate that the value of big data in transfusion medicine cannot be realized without deliberate data integration along AI methods. By addressing heterogeneity, bias, privacy, scalability and interpretability, AI provides a spectrum of solutions that enable data resources to evolve from fragmented silos into interoperable infrastructures.

Following the fundamental introduction into the prospects and shortcomings of the current evolution of the digital transformation in transfusion medicine, we will next exemplify these with practical elements, such as precision RBC product diagnostics or digital crossmatch technologies.

## PRECISION ASSESSMENT OF STORED RED BLOOD CELL QUALITY: INTEGRATING LAB‐ON‐A‐CHIP DIAGNOSTICS WITH BIG DATA AND AI FOR PERSONALIZED TRANSFUSION STRATEGIES

The quality of stored RBCs remains a cornerstone challenge in transfusion medicine, where current practices rely on standardized but simplistic criteria that fail to capture the heterogeneity introduced by donor variability, processing methods, and storage duration [33, 34]. RBC units, stored at 4°C for up to 42 days in additive solutions, undergo progressive biochemical and biophysical changes, known as storage lesions, that can impair oxygen delivery, increase haemolysis risk and reduce post‐transfusion recovery. Traditional quality checks, mandated by regulatory bodies such as the FDA and EMA, focus on endpoint metrics such as <1% haemolysis and >75% 24‐h survival [35] but overlook unit‐specific ‘functional age’ influenced by factors like donor demographics, genetics and lifestyle [33, 36, 37, 38, 39]. These product‐related factors may further aggravate risks, especially in vulnerable populations.

Emerging precision diagnostics, particularly lab‐on‐a‐chip technologies [40, 41], offer a transformative approach to real‐time, unit‐level RBC assessment [33]. These microfluidic platforms miniaturize complex assays into portable, low‐volume devices capable of quantifying storage‐induced changes with high sensitivity and throughput. For instance, lab‐on‐a‐chip systems can evaluate RBC deformability [42, 43, 44, 45], a key indicator of microvascular perfusion [44], by measuring transit times through microconstrictions or assessing shear‐induced elongation under controlled flow [43]. Studies have demonstrated that stiffened RBCs, resulting from storage lesions, can be fractionated based on margination dynamics in laminar flow channels, correlating strongly with osmotic fragility and predicting in vivo performance [46]. Similarly, integrated chemical, optical and electrical sensors in lab‐on‐a‐chip enable rapid non‐destructive detection of haemolysis markers.

The true potential of lab‐on‐a‐chip diagnostics emerges through synergies and convergence with big data and AI, enabling a shift from reactive to predictive, patient‐matched transfusion paradigms [33]. Large‐scale cohorts (e.g., REDS‐III) [38, 47] and the DBDS [5] provide ‘vein‐to‐vein’ datasets integrating donor omics (genomics, metabolomics), processing variables, and recipient outcomes, revealing how genetic polymorphisms (e.g., G6PD) modulate storage lesion severity. ML models, trained on these datasets, can identify predictive biomarkers, such as kynurenine pathway metabolites [20] or oxidative stress indices, that forecast post‐transfusion efficacy beyond storage age.

Through this convergent approach, that is, integrating lab‐on‐a‐chip outputs with AI‐driven comprehensive analytics, transfusion services can create dynamic matching algorithms. Real‐time diagnostic data from inventory units (e.g., deformability scores, haemolysis rates) can feed into federated learning frameworks, where models aggregate insights across blood banks without compromising privacy via techniques like differential privacy. This approach supports personalized allocation, allowing AI to match low‐haemolysis, high‐deformability units to patients with microvascular complications (e.g., sickle cell disease), while directing other units to routine cases, informed by eHR via the FHIR standards. Pilot integrations, such as those linking microfluidic deformability assays with eHR‐derived recipient risk profiles, have demonstrated improved matching, potentially reducing alloimmunization and waste. Moreover, unsupervised clustering of high‐dimensional lab‐on‐a‐chip and omics data can uncover novel lesion subtypes, guiding additive solution optimizations or rejuvenation protocols for further improvements in transfusion outcomes [33]. Several challenges remain on the path towards integrating lab‐on‐a‐chip with AI in transfusion medicine. These include the need for assay standardization, compatibility with legacy blood establishment systems and validation across diverse populations to address biases such as the ‘healthy donor effect’. Current initiatives, such as PETRA, will be pivotal in scaling these tools across borders.

## TWENTY YEARS OF DIGITAL CROSSMATCH RESEARCH: REVISIONS BY AI?

Crossmatching is a cornerstone of transfusion medicine practice, serving as a safety measure prior to the transfusion of RBC concentrates. It includes the ABO blood group typing and antibody screening processes, with the primary aim of preventing haemolytic transfusion reactions that may result from antigen–antibody incompatibilities [48]. Starting in the early 1990s, countries have increasingly replaced the traditional serological crossmatching with the electronic crossmatching (EXM) [49]. The concept of EXM is based on the premise that, in patients without clinically significant antibodies, a computerized system can verify ABO compatibility at least as reliably as a serological test [50], resulting in reduced laboratory workload and greater safety. Following its international success over two decades, the EXM moved from individual exemptions into general use (revised regulation 21 CFR 606.151 FDA) [3, 51]. Furthermore, crossmatch using human leukocyte antigen (HLA) antibody predictions has also proved its clinical utility in solid organ transplantation [52].

In recent years, rapid advances in data integration and AI have created new opportunities to expand the EXM beyond ABO and Rh compatibility to the reliable prediction of additional erythrocyte and human platelet antigens (HPAs). In particular, the increasingly available data from high‐dimensional genotyping is highly informative for predictive models. A landmark example is the study by Hyvärinen et al. [53], which accurately predicted erythrocyte antigens and the HPA 1 from standard whole genome genotyping using random forest models. Initially trained in a Finnish cohort and subsequently validated in a large Danish cohort, the models achieved very high accuracy and underscored the potential of ML‐based methods as a rapid, scalable and precise alternative to serological testing. Given that classical serological techniques for antigen determination are labour‐intensive and resource‐demanding, particularly in the context of large biobanks or rare blood groups, these approaches open promising avenues for the efficient identification of rare antigen‐negative donors and for enhancing transfusion safety [53]. Beyond antigen prediction for precise blood group assignment, the assessment of alloimmunization may also be covered by precision transfusion strategies. Exemplarily, the Minimizing Risk of Alloimmunisation by Assigning Red Blood Cells (MINRAR) approach incorporates not only ABO and Rh but also additional clinically relevant antigens such as Kell and RhCE [54, 55]. By estimating the individual risk of an immune reaction, the system enables more targeted donor recipient allocation and significantly reduces the likelihood of antibody formation.

## EVOLVING TOWARDS AI SUPPORTED, PATIENT‐CENTRED PREDICTIONS OF PLATELET CONCENTRATES: CONSIDERATIONS ON CONSUMPTION, PERSONALIZATION AND IMMUNIZATION

Platelet products are required for bleeding patients with thrombocytopenia or with platelet dysfunction or to prevent bleeding during thrombocytopenia. Unfortunately, platelet products are a rare and expensive source with a short shelf‐life. Several studies have attempted to support donor management by AI [54, 55], or improve donor safety during apheresis procedures by reviewing sources of risk [56, 57]. Such registries may be deployed in a decentralized setting [58] to support haemovigilance. In addition, the fluctuating demand is challenging the supply chains. Several AI tools forecast how much concentrates will be needed [59, 60, 61], which may help adapt the donation or provide products from other blood donation services.

Within the individualized blood supply, platelets apheresis for patients with existing immunization against thrombocyte antigens (e.g., HLA class I and HPAs) is a special challenge. Here, compatibility of patient and donor antigens are critical and it may be difficult to find the suitable product or a donor, depending on the antibody titre or high‐prevalence antigens. However, the reduced costs and availability of next‐generation sequencing allowed to genotype donors for platelet antigens at large scales [62, 63, 64, 65, 66]. Several technology‐driven approaches further responded to this issue: for instance, suitable products tools may be identified by digital crossmatch [67, 68, 69, 70]. This reduces workload, personal resources and cost and increases the corrected count increment. On the level of national initiatives, Hyvärinen K. et al. [53] have extracted HPA‐1 for biobank‐scale genetics in a Finnish blood service biobank and Danish cohort and applied ML. In Greece, the national HPA donor genotype registry was evaluated for the use case of a rare life‐threating newborn disease involving maternal anti‐HPA1 antibodies against foetal or neonatal platelets [71]. Given the low frequency of HPA 1a‐negative donors in the European population (2%), the integration of precision diagnostics, including donor antigen information, strengthens the resilience of blood banks. Most recently, a precision transfusion genotyping chip for the detection of platelet and erythrocyte antigens defined a highly reproducible atlas for extensive matching [56, 57].

## CONCLUSION

Taken together, precision transfusion medicine and diagnostic strategies including lab‐on‐a‐chip, amplified by big data and AI, could usher in an era of individualized transfusions—maximizing efficacy, minimizing risks and optimizing global supply chains. While, a digital workflow is critical for AI incorporation, printouts may accompany products for visualization or as emergency backup. Given its heavy regulation, the evolution of the field will depend on future legal frameworks, while the integration of genomic and clinical data for routine transfusion practice, along with systematic AI systems, can strengthen the safety and quality of transfusions and ideally improve the availability of blood products.

## CONFLICT OF INTEREST STATEMENT

A.T.T. is funded by Neovii Biotech and GTL. He is a consultant for CSL Behring, Maat Pharma, Biomarin, Pfizer and Onkowissen. He gets travel reimbursements from Neovii and Novartis. The other authors declare no conflicts.

## Supporting information


**Table S1.** Practical examples of artificial intelligence (AI) challenges in transfusion medicine and possible solutions.

## Data Availability

Data sharing not applicable to this article as no datasets were generated or analysed during the current study.
