# Supplementary material for: Fundamentals of big data and artificial intelligence in transfusion medicine
Source: Vox Sang. 2026 Mar 23;121(4):430–40. doi: 10.1111/vox.70227 (PMC13066920; doi:10.1111/vox.70227)
Supplement: Supplementary file 1 — Table S1. Practical examples of artificial intelligence (AI) challenges in transfusion medicine and possible solutions. [file VOX-121-430-s001.docx]

**Supplementary material to**

**Fundamentals of Big Data and Artificial Intelligence in Transfusion Medicine**

Amin T. Turki^1,2,3^, Christian Martin Brieske^4^, Umut A Gurkan^5^, Katja M. Scheidler^1, 2^, O. Berk Usta^6^, Esa Turkulainen^7^, Kamyar Arzideh^2^, Christian Temme^4^, René Hosch^2^, Peter A. Horn^4^, and Mikko Arvas^7^

1. Computational Hematology Lab, Institute for Artificial Intelligence in Medicine, University Hospital Essen, Essen, Germany
2. Institute for Artificial Intelligence in Medicine, University Hospital Essen, Essen, Germany
3. Department of Hematology and Oncology, University Hospital Marienhospital, Ruhr-University Bochum, Bochum, Germany
4. Institute for Transfusion Medicine, University Hospital Essen, Essen, Germany
5. Department of Mechanical and Aerospace Engineering, Case Western Reserve University, Cleveland, OH, United States
6. Center for Engineering in Medicine & Surgery, Massachusetts General Hospital, Harvard Medical School, Boston, MA, United States
7. Research and Development, Finnish Red Cross Blood Service, Helsinki, Finnland

Corresponding Author:

Priv.-Doz. Dr. med. Dr. phil. Amin T. Turki, MD/PhD

Computational Hematology Lab, Institute for Artificial Intelligence in Medicine

University Hospital Essen

Hufelandstr. 55, NRW, 45147, Essen

Tel: +49 201-723-0

E-Mail: amin.turki@uk-essen.de

**Supplementary Material**

**Extended explanation of different predictive methods**

**Predictive modelling** uses statistical and machine learning methods to forecast outcomes such as disease progression, remission, or transfusion-related complications, enabling early intervention and personalized care. While powerful for risk stratification, predictive models depend on data quality and completeness and may reproduce biases from underrepresented populations. They also raise concerns about privacy, technical integration into hospital IT, and the “black box” opacity of many algorithms. **Rule-based decision support** systems rely on predefined clinical guidelines and logical rules grounded in evidence-based medicine. They are transparent and intuitive, embedding knowledge that clinicians already use, which fosters trust and adherence. However, they are inflexible, require clean inputs, and must be continuously updated by domain experts to remain current. In transfusion medicine, examples include electronic crossmatch protocols and standardised hemovigilance alerts. **Supervised learning** is the most widely applied machine learning paradigm, where models predict labelled outcomes by estimating conditional probabilities p(Y|X). Techniques include decision tree–based random forests, stacked ensemble models, regularised regression approaches such as LASSO, and Bayesian regression methods. Applications in transfusion medicine include predicting hemoglobin increments or complications following allogeneic HCT. **Unsupervised learning** does not rely on predefined labels but instead identifies patterns and structures within high-dimensional data. Approaches include clustering and dimensionality reduction (e.g., PCA, UMAP). These methods are particularly useful for integrating heterogeneous data such as multi-omics, immune profiling, and eHRs, though they are subject to the “curse of dimensionality,” where distances between data points lose discriminative power. Clusters are often validated with survival analysis to link exploratory findings back to outcomes. **Reinforcement learning** is a paradigm in which algorithms learn through sequential interactions with their environment, receiving feedback in the form of rewards or penalties. Although rarely applied in hematology to date, it could be used in transfusion medicine to optimize platelet inventory redistribution across networks in real time. **Deep learning** architectures extend machine learning through >1 hidden layers, enabling highly flexible representations. Neural networks, natural language processing (NLP) pipelines, and transformers underpin recent progress in unstructured data analysis, including free-text notes, imaging, and omics. These models form the basis for **large language models (LLMs)**, which combine predictive accuracy with structured reasoning, produce natural-language explanations, and personalise recommendations according to user expertise or workflow. However, they are prone to hallucinations or confabulations and must be safeguarded through grounding in validated data and human oversight.

###### **Supplementary Tables**

**Supplementary Table 1: Practical examples of AI Challenges in Transfusion Medicine and possible solutions**

| **Challenge** | **Category** | **Examples of possible solutions** |
| --- | --- | --- |
| Fragmented data across eHR/ donor registries/ BECS | Different identifiers; free-text events | ISBT-128 codes in the warehouse; FHIR profiles for products/procedures; entity resolution. |
| Sparse labels for AEs | Under-reporting; few adjudicated cases | Natural language processing to surface candidates; weak supervision; semi-supervised learning. |
| Small sites, privacy barriers | Can’t share row-level data | Federated learning across hospitals/blood services. |
| Drift (practice, donors, assays) | Forecasts decay; models stale | Rolling backtesting; drift monitors; human-in-the-loop questions and answer pairs |
| Decision accountability | Clinical acceptance | Transparent rules + model cards; prospective audits; AABB/FDA alignment. |

Abbreviations: FDA, food and drug administration, AABB, Association for the Advancement of Blood & Biotherapies, eHR, electronic health records; AE, adverse events.
